# Evaluation of house staff candidates for program fit: a cohort-based controlled study

**DOI:** 10.1186/s12909-022-03801-0

**Published:** 2022-11-01

**Authors:** Soo-Hoon Lee, Phillip H. Phan, Sanjay V. Desai

**Affiliations:** 1 Strome College of Business, Old Dominion University, Norfolk, VA USA; 2 Johns Hopkins Carey Business School, Johns Hopkins University, Baltimore, MD USA; 3 Johns Hopkins School of Medicine, Johns Hopkins University, Baltimore, MD USA

**Keywords:** Housestaff recruitment, Behaviorally Anchored Rating Scale, Quasi-experiment, ACGME Milestones, Longitudinal study

## Abstract

**Background:**

Medical school academic achievements do not necessarily predict house staff job performance. This study explores a selection mechanism that improves house staff-program fit that enhances the Accreditation Council for Graduate Medical Education Milestones performance ratings.

**Objective:**

Traditionally, house staff were selected primarily on medical school academic performance. To improve residency performance outcomes, the Program designed a theory-driven selection tool to assess house staff candidates on their personal values and goals *fit* with Program values and goals. It was hypothesized cohort performance ratings will improve because of the intervention.

**Methods:**

Prospective quasi-experimental cohort design with data from two house staff cohorts at a university-based categorical Internal Medicine Residency Program. The intervention cohort, comprising 45 house staff from 2016 to 2017, was selected using a Behaviorally Anchored Rating Scales (BARS) tool for program fit. The control cohort, comprising 44 house staff from the prior year, was selected using medical school academic achievement scores. House staff performance was evaluated using ACGME Milestones indicators. The mean scores for each category were compared between the intervention and control cohorts using Student’s t-tests with Bonferroni correction and Cohen’s d for effect size.

**Results:**

The cohorts were no different in academic performance scores at time of Program entry. The intervention cohort outperformed the control cohort on all 6 dimensions of Milestones by end-PGY1 and 3 of 6 dimensions by mid-PGY3.

**Conclusion:**

Selecting house staff based on compatibility with Residency Program values and objectives may yield higher job performance because trainees benefit more from a better fit with the training program.

**Supplementary Information:**

The online version contains supplementary material available at 10.1186/s12909-022-03801-0.

## Introduction

Selecting house staff candidates takes time and considerable resources. Program directors for medical and surgical residency programs often rely on cognitive examination-based metrics, such as the United States Medical Licensing Examination (USMLE) scores, and other academic metrics, such as Alpha Omega Alpha (AOA) Honor Medical Society membership, to select program candidates [1, 2]. However, the effectiveness of such metrics to predict the performance of candidates when they become house staff has been mixed across various disciplines in multicenter studies. Some studies found modest correlations between academic metrics with subsequent faculty performance ratings [3, 4], while others found no relationship [5, 6]. Specifically, USMLE scores only predict residents’ medical knowledge, in-training and board examination scores but do not correlate with faculty ratings on residents’ clinical judgment and acumen, clinical skill acquisition, patient rapport, work ethic, or resident ranking at the time of graduation in a multicenter study and in various disciplines [3, 4, 7–9]. Being a “great” resident requires other qualities such as being personable or professionalism besides academic achievement that are assessed during selection interviews [10, 13]. Interviews are used to capture non-cognitive qualities, such as interpersonal and communication skills, that are valued and emphasized by a variety of residency programs [2, 10–12]. While structured interviews, in which all interviewees are asked the same questions, are more reliable and less biased than unstructured interviews, their ability to predict resident performance has been weak or non-significant [14, 15]. The limitations from using academic metrics and structured interviews points to a research gap. Drawing from the selection literature, we consider how an interview *scoring tool* could improve the selection of house staff. We hypothesize that interviewees who are evaluated on rubrics that are explicitly anchored to organizational values will outperform those who are not similarly evaluated and selected.

We base our hypothesis on two organizational concepts that suggest a way to improve the *evaluation* of house staff candidates. The first is person-organization fit [16], which posits that the congruence of values and goals between the individual and organization enhances positive attitudes and work behaviors. The second is attraction-selection-attrition (ASA) theory [17], which explains that individuals are attracted to organizations with similar values, and likewise, organizations select candidates who are most likely to assimilate their cultures. Matching people with their organization’s culture enhances work performance and satisfaction [18–20]. Conversely, individuals who do not match on these dimensions may not perform as well, and eventually leave or are let go.

To test our hypothesis, we assess a cohort of house staff on the fit between their professional and personal attitudes and values with values and objectives of the Program during the interview. We use a behaviorally anchored rating scale (BARS) tool, together with blinded interviewers, to improve intra- and inter-rater reliability, during the evaluation phase of the house staff interviewing process [21]. In the BARS intervention, interviewees are evaluated by a set of criteria based on values and objectives that are important to the Program (see Supplemental Appendix 1 for samples of BARS). The rating scales for each criterion are anchored to behavioral exemplars, narratives, or critical incidents that represent specific levels of performance success as required by the Program for each criterion. We compare the subsequent performance of house staff selected with BARS against a control cohort selected in the prior year without BARS. We test if house staff evaluated on BARS during the interview outperforms the control cohort in the residency program.

## Methods

### Study design, population, and settings

This quasi-experimental prospective study employs data from two cohorts of house staff at a university-based categorical Internal Medicine Residency Program. Both cohorts had previously been shortlisted for interviews based on academic qualifications and extra-curricular experiences. Both cohorts were interviewed twice by two different interviewers during the site visit. The INTERVENTION cohort comprised all 45 house staff selected from 323 applicants who interviewed between November 2016 and January 2017. 97 faculty from across the Department of Medicine participated in the interviews. Interviewers were asked to use the BARS scoring rubric in which 1 (high), 3 (average), and 5 (low) ratings for each criterion were anchored to exemplar behaviors, narratives, or critical incidents critical to the values and objectives of the Residency Program for that criterion [22, 23]. The first interviewer asked questions on criteria related to clinical activities and scholarly research. Candidates discussed their experiences in medical school that were ‘meaningful, challenging, or great’ in these domains. The second interviewer evaluated candidates on the leadership criterion. Candidates described the opportunities they would ‘improve upon, look forward to doing, and strive in’ to advance their career as a leader. To match the way interviews are conducted in real time, interviewers were given latitude in how they presented the questions. Using BARS ensure rating consistencies between interviewers. Interviewers submitted their completed BARS scores by email to the Director of the Residency Program within a week of each interview. From the scores, internal discussions by the Intern Selection Committee were held for applicants who tied on the BARS scores. Each applicant was subsequently given an interview ranking based on their BARS scores and from the internal discussions.

The CONTROL cohort comprised all 44 house staff selected from 404 applicants who interviewed between November 2015 and January 2016. Interviewers evaluated the applicants using an overall single numerical score between 1 and 10, with 1 as the highest. Interviewers were drawn from approximately the same pool for the two cohorts except those who left the institution or were new hires. The latter set comprised less than 5% of the interviewer pool.

The Institutional Review Committee approved this study and data were deidentified.

### Data collection and quality control

Interviewer-candidate pairs were randomly assigned. To minimize the Hawthorne and other selection biases, interviewers were blinded to the study’s objective. To standardize the intervention and to ensure complete data collection, interviewers were given a set of questions and the scoring rubric. Subsequently, house staff annual performances were evaluated by 12 standing members of the faculty from the Clinical Competency Committee (CCC) who use the Accreditation Council for Graduate Medical Education (ACGME) Milestones Performance indicators [24, 25]. Members of the CCC were selected for their experience in performance evaluation, regular interactions with the house staff, and knowledge of the house staff’s performance in clinical settings and interactions with co-workers.

### Outcome measures

House staff performance for both CONTROL and INTERVENTION groups was evaluated on 22 items in Milestones Performance comprising six core competency areas: Patient Care (PC) includes 5 items that measure physicians’ taking a patient-centered approach to healthcare; Medical Knowledge (MK) includes 2 items that measure physicians’ ability to apply medical knowledge to clinical situations; Systems-based Learning (SBL) includes 4 items that measure physicians’ ability to coordinate patient care within the healthcare system; Practice-based Learning (PBL) includes 4 items that measure physicians’ commitment to lifelong learning; Professionalism (PROF) includes 4 items that measure physicians’ treating all people with respect, compassion, and dignity; and Interpersonal Communication (IC) includes 3 items that measure physicians’ ability to effectively exchange information with patients, their families, and professional associates [24, 25]. Milestones Performance data was analyzed at PGY1 mid-year, PGY1 year-end, and PGY3 mid-year. PGY2 data was not analyzed because in this program, house staff rotate out of the Internal Medicine Department to other disciplines, so members of the CCC could not directly observe the house staff performance on a regular basis. PGY3 year-end data was not measured because house staff were interviewing for fellowships and jobs, reducing opportunities for the CCC to observe performance. Milestones Performance were scored from 1 (poor) to 9 (exceptional) on each item. We calculated a mean score for each of the six Milestones Performance core competencies for each house staff.

### Intervention and measures

All house staff’s academic performance were collected at the time of the interview and included as covariates. These comprised USMLE Step 1 and Step 2 scores, which proxy the measurement of clinical knowledge, AOA membership (1 = Yes, 0 = No), which recognizes performance representing the top 10% of the graduating medical school class, and medical school ranking listed in the US News and World Report. The fifth covariate was each house staff’s interview ranking.

The INTERVENTION comprises the cohort of house staff selected on Program fit using a BARS scoring rubric, coded as 1. The CONTROL comprises the cohort of house staff selected on academic performance, coded as 0. Intervention subjects were selected on behavioral and attitudinal fit with the Program’s clinical, scholarly research, and leadership task expectations, values, and an overall assessment on potential success as a house staff in the Program. The BARS scoring rubric consisted of 5-point rating scales in which ratings of ‘1’, ‘3’, and ‘5’ were anchored to exemplar behaviors for each criterion.

Specifically, a criterion and Program value is *clinical competency*, which is the ability to apply theoretical knowledge to clinical practice. House staff are expected to take responsibility for their patients and make clinical decisions when assessing patients and performing procedures. Another criterion and Program value is self-directed learning and *scholarly research*. House staff are expected to conduct original research and publish papers to contribute to the general body of medical knowledge. Engagement in research provides house staff with opportunities to acquire knowledge to handle novel and unpredictable situations as well as be thought leaders in specialty domains [10]. A third is grooming *leaders* to meet challenges in quality, safety, and patient-centered care [12]. Candidates are evaluated on their potential for leadership in innovation, quality improvement, or community engagement. *Program fit* and *potential success* measure candidates’ attitudinal fit to the Program’s values and their potential to succeed in the Program respectively.

### Statistical analysis

To determine if the house staff in the INTERVENTION and CONTROL cohorts were comparable, medical school achievement at the start of the Residency Program were evaluated for differences using Student’s t-tests. To test the hypothesis that the INTERVENTION was associated with higher performance, we compared the Milestones Performance scores between the INTERVENTION and CONTROL cohorts using Student’s t-tests. Bonferroni correction was applied to adjust the statistical significance threshold for multiple comparisons and Cohen’s d was applied to determine the effect size of the differences or the practical significance of the outcomes between the two groups.

To further test the hypothesis that the intervention was associated with house staff performance, hierarchical regression analysis was performed in which their academic and interview rankings were first entered into the regression model, followed by the binary Intervention variable. We report the standardized regression coefficients or beta, which allows for easier comparisons among variables that are measured in different units or dimensions to standardize the relationship with the outcome variable. Statistical significance is set at p < 0.05 and with the Bonferroni correction threshold at p = 0.008. A statistical significance on INTERVENTION indicates that an interview process that evaluates applicants’ clinical, research, and leadership behavioral and attitudinal fit, fit with Program values, and overall potential success in the Program not only shows significant differences between the cohorts but also add incremental prediction on performance beyond factors related to academics and interview ranking.

## Results

Total possible number of participants was 45 for the INTERVENTION and 44 for the CONTROL, which were the entire class of respective cohorts. Actual number of participants was 45 for the INTERVENTION and 44 for the CONTROL for a response rate of 100% for both cohorts. Table 1 reports the t-tests between the CONTROL and INTERVENTION cohorts’ academic and interview performance at the time they applied to the Residency Program. The data shows that the two cohorts were not significantly different in terms of their USMLE Step 1 (245.18 ± 15.34 v 247.53 ± 13.38, p = 0.47) and Step 2 scores (250.74 ± 21.54 v 258.18 ± 10.67, p = 0.07), medical school ranking (12.05 ± 17.74 v 9.51 ± 11.43, p = 0.43), or interview rankings (137.47 ± 75.40 v 139.73 ± 79.85, p = 0.89). However, 57.78% of the INTERVENTION cohort had AOA membership, which was significantly higher than 31.82% of the CONTROL cohort (p = 0.01).

**Table 1:** Residents’ Academic and Interview Performance at the Time of Application

Performance Dimensions	Group	n	Mean	s.d.	p-value^*^	Cohen’s d^**^
USMLE Step 1 Score	CONTROL Cohort	44	245.18	15.34		
	INTERVENTION Cohort	44	247.43	13.38	0.47	0.15
USMLE Step 2 Score	CONTROL Cohort	34	250.74	21.54		
	INTERVENTION Cohort	38	258.18	10.67	0.07	0.35
AOA Membership	CONTROL Cohort	44	0.32	0.47		
	INTERVENTION Cohort	45	0.58	0.50	0.01	1.89
Medical School Ranking	CONTROL Cohort	42	12.05	17.74		
	INTERVENTION Cohort	45	9.51	11.43	0.43	0.21
Interview Ranking	CONTROL Cohort	43	137.47	75.40		
	INTERVENTION Cohort	44	139.73	79.85	0.89	0.02

s.d. = standard deviation

^*^Bonferroni corrected threshold for significance p = 0.01

^**^Cohen’s d < 0.1 (trivial effect size); Cohen’s d between 0.1–0.3 (small effect size); Cohen’s d between 0.3–0.5 (moderate effect size) Cohen’s d > 0.5 (large effect size)^26^

Table 2 reports the t-tests between the CONTROL and INTERVENTION cohorts on six core competencies of Milestones Performance at PGY1 Mid-year, PGY1 Year-end, and PGY3 Mid-year. 6 months after the start of the Residency Program (PGY1 Mid-year), the INTERVENTION and CONTROL cohorts are relatively similar in terms of performance. At that time, the INTERVENTION group only outperformed the CONTROL cohort on one Milestones Performance core competency pertaining to PBL (5.72 ± 1.01 v 5.21 ± 0.86, p = 0.01) on their commitment to lifelong learning. By the end of PGY1 (PGY1 Year-end), the INTERVENTION group outperformed the CONTROL group on five Milestones Performance core competencies except for MK. Near the end of PGY3 (PGY3 Mid-year), the INTERVENTION group outperformed the CONTROL cohort on all six Milestones Performance core competencies. Detailed differences on 22 items of Milestones Performance are shown in Supplemental Appendix 2. Finally, we note that the effect sizes for the performance differences were not only statistically significant after multiple comparison corrections, but also practically meaningful, as indicated by the Cohen’s d scores.

**Table 2:** Comparison on Milestones Performance Core Competencies

	CONTROL Cohort	INTERVENTION Cohort	p-value^*^	Cohen’s d^**^
**PGY1 Mid-year Performance Evaluation**	**Mean ± s.d.**	**Mean ± s.d.**		
Patient Care (PC)	4.46 ± 0.80	4.71 ± 0.47	0.07	0.31
Medical Knowledge (MK)	4.55 ± 0.75	4.74 ± 0.63	0.18	0.25
Systems-Based Practice (SBP)	5.16 ± 0.82	5.14 ± 0.69	0.90	0.02
Practice-Based Learning (PBL)	5.21 ± 0.86	5.72 ± 1.01	0.01	0.59
Professionalism (PROF)	5.33 ± 0.74	5.71 ± 1.12	0.07	0.51
Interpersonal Communication (IC)	5.38 ± 0.81	5.64 ± 1.00	0.18	0.32
**PGY1 Year-End Performance Evaluation**				
Patient Care (PC)	4.76 ± 0.68	5.15 ± 0.45	0.002*	0.57
Medical Knowledge (MK)	4.89 ± 0.74	5.10 ± 0.33	0.08	0.28
Systems-Based Practice (SBP)	5.14 ± 0.87	5.74 ± 0.76	0.001*	0.69
Practice-Based Learning (PBL)	5.25 ± 0.92	6.03 ± 0.83	< 0.001*	0.85
Professionalism (PROF)	5.32 ± 0.94	6.21 ± 1.20	< 0.001*	0.95
Interpersonal Communication (IC)	5.40 ± 0.88	6.42 ± 0.89	< 0.001*	1.16
**PGY3 Mid-year Performance Evaluation**				
Patient Care (PC)	7.03 ± 0.67	7.54 ± 0.62	0.001*	0.76
Medical Knowledge (MK)	7.22 ± 0.84	7.62 ± 0.73	0.02	0.48
Systems-Based Practice (SBP)	7.35 ± 0.63	7.55 ± 0.55	0.13	0.32
Practice-Based Learning (PBL)	7.14 ± 0.78	7.58 ± 0.64	0.01	0.34
Professionalism (PROF)	7.34 ± 0.80	7.94 ± 0.80	0.001*	0.75
Interpersonal Communication (IC)	7.33 ± 0.75	7.82 ± 0.68	0.002*	0.65

**s.d.** = standard deviation

Bonferroni corrected threshold for significance p = 0.008

^*^statistically significant at Bonferroni threshold

^**^Cohen’s d < 0.1 (trivial effect size); Cohen’s d between 0.1–0.3 (small effect size); Cohen’s d between 0.3–0.5 (moderate effect size) Cohen’s d > 0.5 (large effect size)^26^

Since the INTERVENTION cohort had higher AOA membership scores, we include medical school academic performance covariates in the regression analysis to control for potential bias between the two groups. Table 3 shows that after controlling for the house staff’s academic performance at the time of entry in the Program, the INTERVENTION cohort was not significantly different from the CONTROL cohort in Milestones Performance 6 months into the Program. However, by the end of PGY1 and by the middle of PGY3, the INTERVENTION cohort reported significantly higher Milestones Performance compared to the CONTROL cohort.

**Table 3:** Hierarchical Regression of the Intervention on Milestones Performance over Time

	PGY1 Mid-year Milestones Performance	PGY1 Year-end Milestones Performance	PGY3 Mid-year Milestones Performance
*Block 1*	Beta	Beta	Beta
USMLE Step 1 Score	0.39*	0.28	-0.04
USMLE Step 2 Score	-0.21	0.01	0.17
AOA Membership	0.03	-0.16	-0.06
Medical School Ranking	-0.09	-0.22^*^	-0.02
Interview Ranking	0.09	0.05	0.13
*Block 2*			
INTERVENTION Cohort	0.08	0.43^***^	0.38^**^

Beta = standardized regression coefficient

^*^p < 0.05

^**^p < 0.01

^***^p < 0.001

## Discussion

While USMLE Step 1 scores had a significant influence on faculty evaluations 6 months into the Program, academic achievements from medical schools had no significant influence by the middle of PGY3, which is consistent with past results [3, 7–9]. Instead, the cohort of house staff selected on fit with Program’s values and objectives had higher performance at the end of their residency period compared to those selected on medical school academic performance. The results are consistent with predictions and explanations from person-organization fit and ASA theories that individuals who are matched to organizations with similar values have higher work performance [18–20]. The results suggest that house staff who have compatible values and goals with Program values and objectives may have higher performance in programs that emphasize those values in the training curriculum.

The interviewers from both cohorts were drawn from the same pool of attending physicians, the Program curriculum did not change between the INTERVENTION and CONTROL cohorts, and the performance evaluators and Milestones Performance dimensions were the same between the cohorts. The difference, after accounting for individual candidate differences at time of entry, was the criteria in which the candidates were selected. Instead of selecting applicants on academic performance, explicit evaluation of applicants using a BARS rubrics based on fit with Program’s values and objectives that considered non-cognitive dimensions such as leadership, clinical skill acquisition, and research work ethic was associated with higher performance, which is consistent with other studies that highlight such dimensions that make for a ‘great’ resident [3, 4, 7–9]. The sustained Milestones Performance after more than two years suggest that the selection method is sustainable over the training period. However, creating an interview process around Program fit requires selecting criteria related to Program values and objectives as well as an explicit scoring rubric. We find that the validity and reliability of the interview process, as evidenced by subsequent candidate performance, was improved when using BARS tool, where the criteria are related to the Program values and objectives, and rating for each criterion are anchored to exemplar behaviors, attitudes, and values.

With respect to the implications of our results in the context of previous studies, one of the most interesting findings from previous studies in internal medicine and other disciplines, is the weak relationship between the use of structured interviews and subsequent house staff performance. On the one hand, this is surprising because one would expect, *prima facie*, that structured interviews reduce bias. On the other hand, structured interviews that do not have an explicit scoring rubric for interviewees’ responses may not improve the predictability of the candidate’s performance. Our study neatly closes this gap with the BARS tool because it quantifies the assessment of the candidates’ interview responses.

A potential limitation is the unequal restriction of range in interview scores (25 points in the INTERVENTION and 10 in the CONTROL). Although we did not use the data to compare the selection committee’s scores across groups, we are unable to ascertain the degree of unobserved bias, if it exists in the selection process. For example, a larger range of 25 points from the BARS scores could result in a smaller number of ties which will require the selection committee to make a decision that is not strictly based on the scoring. Even though we evaluated the entire class for each cohort, the statistical power of the sample is likely to be small. In our study, this is a strength since, despite the small sample, the *consistent* statistical significance of the performance differences reveals the strength of the Intervention.

Another limitation is that we cannot ‘prove’ that program compatibility was better established in the intervention cohort than the control during the interviewing stage of the selection process. However, we know that the data on compatibility in the INTERVENTION cohort is more consistent because the scoring rubrics in the BARS tool ensures that all the interviewers rated the interviewees on rubrics that reflect the values of the Program. As well, the selection committee used these data to make their selection decisions. While interviewers in the CONTROL cohort may have asked questions related to program compatibility, we do not have the same degree of confidence that everyone did so, because they were not explicitly told to evaluate interviewees’ responses on Program values.

## Conclusion

Although this is a single center study, it makes sense that the approach to selecting house staff on program fit could be generalized since every training program has specific learning objectives and assessments of learning criteria. The key to standardizing the selection process is to minimize unwanted variation, which was the purpose of the BARS tool. An obvious extension would be to replicate the BARS development procedure in more sites, using the ACGME Milestones Performance criteria as a standard outcome measure. We also recognize that medical education is constantly evolving, so recent cohorts could naturally outperform prior cohorts over time. We have no evidence from the literature that this is happening but acknowledge the possibility. Finally, it would be easy to attribute the results to the fact that this study was conducted in an elite residency program. However, the intervention is designed to be extensible and adaptable to any training program because it is based on a selection mechanism that improves the match between candidates and program values and objectives. The lesson from this intervention is for program directors to clearly translate their programs’ values and objectives, which can be challenging, to measurable dimensions on which interview data can be judged against.

## Electronic supplementary material

Below is the link to the electronic supplementary material.

Supplementary Material 1

## Data Availability

Under the Family Educational Rights and Privacy Act (FERPA) (20 U.S.C. § 1232 g; 34 CFR Part 99), the raw data cannot be released. However, summary data is available from the first author upon reasonable request.

## List of abbreviations

ACGME: Accreditation Council for Graduate Medical Education.
AOA: Alpha Omega Alpha Honor Medical Society.
ASA: Attraction-Selection-Attrition theory.
BARS: Behaviorally Anchored Rating Scales.
CCC: Clinical Competency Committee.
IC: Interpersonal Communication (Milestones dimension).
MK: Medical Knowledge (Milestones dimension).
PBL: Practice-based Learning (Milestones dimension).
PC: Patient Care (Milestones dimension).
PROF: Professionalism (Milestones dimension).
SBL: Systems-based Learning (Milestones dimension).
USMLE: United States Medical Licensing Examination.
