# Supplementary material for: Evaluation of house staff candidates for program fit: a cohort-based controlled study
Source: BMC Med Educ. 2022 Nov 1;22:754. doi: 10.1186/s12909-022-03801-0 (PMC9628087; doi:10.1186/s12909-022-03801-0)
Supplement: Supplementary file 1 — Supplementary Material 1 [file 12909_2022_3801_MOESM1_ESM.docx]

**Supplemental Material**

**Appendix 1: Sample of BARS on Leadership and Scholarship Performance Dimensions of Selection Criteria**

**Assessment Leadership Performance**:

Score Anchors

1. President, Leader, or Founder of an Organization

2

3 Meaningful role or impact on an Organization

4

5 Membership only in Organizations

**Scholarship Performance**:

Score Anchors

1. Multiple peer reviewed pubs; 1yr or more of dedicated research time

2

3 Meaningful research experiences without substantial productivity

4

5 Little exposure to research experiences; no productivity

**Appendix 2: Comparison on Detailed Milestones Performance**

|  | **CONTROL Cohort** | **INTERVENTION Cohort** | **p-value*** | **Cohen’s d**** |
| --- | --- | --- | --- | --- |
| **PGY1 Mid-year Performance Evaluation** |  |  |  |  |
| Patient Care: Gathers & synthesizes essential & accurate info to define each patient's clinical problems (PC1) | 4.34 ± 0.91 | 4.62 ± 0.58 | 0.09 | 0.31 |
| Patient Care: Develops and achieves comprehensive management plan for each patient (PC2) | 4.39 ± 0.90 | 4.47 ± 0.73 | 0.64 | 0.07 |
| Patient Care: Manages patients with progressive responsibility and independence (PC3) | 4.55 ± 0.88 | 4.62 ± 0.81 | 0.69 | 0.08 |
| Patient Care: Skill in performing procedures (PC4) | 4.83 ± 0.98 | 4.93 ± 0.25 | 0.81 | 0.10 |
| Patient Care: Requests & provides consultative care (PC5) | 4.52 ± 0.79 | 4.93 ± 0.54 | 0.01 | 0.52 |
| Medical Knowledge: Clinical knowledge (MK1) | 4.50 ± 0.76 | 4.71 ± 0.73 | 0.19 | 0.28 |
| Medical Knowledge: Knowledge of diagnostic testing & procedures (MK2) | 4.56 ± 0.80 | 4.78 ± 0.64 | 0.16 | 0.28 |
| Systems-Based Practice: Works effectively within an interprofessional team (SBP1) | 5.18 ± 1.17 | 5.53 ± 1.14 | 0.15 | 0.29 |
| Systems-Based Practice: Recognizes system error & advocates for system improvement (SBP2) | 5.30 ± 0.80 | 5.02 ± 0.78 | 0.11 | 0.35 |
| Systems-Based Practice: Identifies forces that impact the cost of healthcare & advocates for & practices cost-effective care (SBP3) | 5.05 ± 0.75 | 4.89 ± 0.57 | 0.27 | 0.21 |
| Systems-Based Practice: Transitions patients effectively within & across health delivery systems (SBP4) | 5.14 ± 0.93 | 5.13 ± 0.79 | 0.99 | 0.01 |
| Practice-Based Learning: Monitors practice with a goal for improvement (PBLI1) | 5.36 ± 0.97 | 5.84 ± 1.15 | 0.04 | 0.49 |
| Practice-Based Learning: Learns & improves via performance audit (PBLI2) | 5.09 ± 0.86 | 5.67 ± 1.07 | 0.01 | 0.67 |
| Practice-Based Learning: Learns & improves via feedback (PBLI3) | 5.39 ± 1.08 | 5.89 ± 1.19 | 0.04 | 0.46 |
| Practice-Based Learning: Learns & improves at the point of care (PBLI4) | 5.00 ± 0.86 | 5.47 ± 0.97 | 0.02 | 0.55 |
| Professionalism: Has professional & respectful interactions with patients, caregivers & members of the interprofessional team (PROF1) | 5.50 ± 1.02 | 5.93 ± 1.32 | 0.09 | 0.42 |
| Professionalism: Accepts responsibility & follow through on tasks (PROF2) | 5.30 ± 0.95 | 5.71 ± 1.31 | 0.09 | 0.43 |
| Professionalism: Responds to each patient's unique characteristics & needs (PROF3) | 5.20 ± 0.73 | 5.38 ± 1.07 | 0.38 | 0.25 |
| Professionalism: Exhibits integrity & ethical behavior in professional conduct (PROF4) | 5.32 ± 0.86 | 5.80 ± 1.27 | 0.04 | 0.56 |
| Interpersonal Communication: Communicates effectively with patients & caregivers (ICS1) | 5.27 ± 0.73 | 5.71 ± 1.24 | 0.05 | 0.60 |
| Interpersonal Communication: Communicates effectively with interprofessional teams (ICS2) | 5.43 ± 1.04 | 5.80 ± 1.20 | 0.13 | 0.36 |
| Interpersonal Communication: Appropriate Utilization & completion of health records (ICS3) | 5.43 ± 0.97 | 5.40 ± 0.89 | 0.87 | 0.03 |
|  |  |  |  |  |
| **PGY1 Year-End Performance Evaluation** |  |  |  |  |
| Patient Care: Gathers & synthesizes essential & accurate info to define each patient's clinical problems (PC1) | 4.70 ± 0.82 | 5.02 ± 0.50 | 0.03 | 0.39 |
| Patient Care: Develops and achieves comprehensive management plan for each patient (PC2) | 4.84 ± 0.75 | 5.00 ± 0.43 | 0.22 | 0.21 |
| Patient Care: Manages patients with progressive responsibility and independence (PC3) | 4.80 ± 0.79 | 5.02 ± 0.40 | 0.10 | 0.28 |
| Patient Care: Skill in performing procedures (PC4) | 4.61 ± 0.65 | 5.25 ± 0.58 | 0.001* | 0.98 |
| Patient Care: Requests & provides consultative care (PC5) | 4.84 ± 0.68 | 5.51 ± 0.73 | <0.001* | 0.99 |
| Medical Knowledge: Clinical knowledge (MK1) | 4.93 ± 0.87 | 5.09 ± 0.36 | 0.27 | 0.18 |
| Medical Knowledge: Knowledge of diagnostic testing & procedures (MK2) | 4.84 ± 0.78 | 5.11 ± 0.32 | 0.04 | 0.35 |
| Systems-Based Practice: Works effectively within an interprofessional team (SBP1) | 5.21 ± 0.93 | 6.00 ± 1.04 | <0.001* | 0.85 |
| Systems-Based Practice: Recognizes system error & advocates for system improvement (SBP2) | 5.11 ± 0.97 | 5.62 ± 0.89 | 0.01 | 0.53 |
| Systems-Based Practice: Identifies forces that impact the cost of healthcare & advocates for & practices cost-effective care (SBP3) | 5.11 ± 0.84 | 5.60 ± 0.78 | 0.01 | 0.58 |
| Systems-Based Practice: Transitions patients effectively within & across health delivery systems (SBP4) | 5.14 ± 0.90 | 5.76 ± 5.76 | 0.001* | 0.69 |
| Practice-Based Learning: Monitors practice with a goal for improvement (PBLI1) | 5.39 ± 0.99 | 6.16 ± 0.85 | <0.001* | 0.78 |
| Practice-Based Learning: Learns & improves via performance audit (PBLI2) | 5.16 ± 0.96 | 5.91 ± 0.97 | <0.001* | 0.78 |
| Practice-Based Learning: Learns & improves via feedback (PBLI3) | 5.39 ± 1.08 | 6.04 ± 0.95 | 0.003 | 0.60 |
| Practice-Based Learning: Learns & improves at the point of care (PBLI4) | 5.07 ± 0.95 | 6.02 ± 0.81 | <0.001* | 1.00 |
| Professionalism: Has professional & respectful interactions with patients, caregivers & members of the interprofessional team (PROF1) | 5.39 ± 0.95 | 6.24 ± 1.54 | 0.002* | 0.89 |
| Professionalism: Accepts responsibility & follow through on tasks (PROF2) | 5.21 ± 1.09 | 6.18 ± 1.19 | <0.001* | 0.89 |
| Professionalism: Responds to each patient's unique characteristics & needs (PROF3) | 5.27 ± 0.97 | 6.04 ± 1.09 | 0.001* | 0.79 |
| Professionalism: Exhibits integrity & ethical behavior in professional conduct (PROF4) | 5.43 ± 1.11 | 6.38 ± 1.43 | 0.001* | 0.86 |
| Interpersonal Communication: Communicates effectively with patients & caregivers (ICS1) | 5.36 ± 0.92 | 6.36 ± 1.07 | <0.001* | 1.09 |
| Interpersonal Communication: communicates effectively with interprofessional teams (ICS2) | 5.39 ± 0.99 | 6.42 ± 1.03 | <0.001* | 1.04 |
| Interpersonal Communication: Appropriate Utilization & completion of health records (ICS3) | 5.46 ± 1.02 | 6.49 ± 0.73 | <0.001* | 1.01 |
|  |  |  |  |  |

| **PGY3 Mid-year Performance Evaluation** |  |  |  |  |
| --- | --- | --- | --- | --- |
| Patient Care: Gathers & synthesizes essential & accurate info to define each patient's clinical problems (PC1) | 7.27 ± 0.95 | 7.61 ± 0.83 | 0.09 | 0.36 |
| Patient Care: Develops and achieves comprehensive management plan for each patient (PC2) | 7.07 ± 1.03 | 7.68 ± 0.82 | 0.004 | 0.59 |
| Patient Care: Manages patients with progressive responsibility and independence (PC3) | 7.17 ± 0.77 | 7.63 ± 0.73 | 0.01 | 0.60 |
| Patient Care: Skill in performing procedures (PC4) | 6.34 ± 0.53 | 7.24 ± 0.54 | <0.001* | 1.70 |
| Patient Care: Requests & provides consultative care (PC5) | 7.32 ± 0.69 | 7.54 ± 0.67 | 0.15 | 0.32 |
| Medical Knowledge: Clinical knowledge (MK1) | 7.20 ± 0.87 | 7.63 ± 0.77 | 0.02 | 0.49 |
| Medical Knowledge: Knowledge of diagnostic testing & procedures (MK2) | 7.24 ± 0.86 | 7.61 ± 0.74 | 0.04 | 0.43 |
| Systems-Based Practice: Works effectively within an interprofessional team (SBP1) | 7.46 ± 0.78 | 7.66 ± 0.76 | 0.26 | 0.26 |
| Systems-Based Practice: Recognizes system error & advocates for system improvement (SBP2) | 7.32 ± 0.82 | 7.46 ± 0.55 | 0.35 | 0.17 |
| Systems-Based Practice: Identifies forces that impact the cost of healthcare & advocates for & practices cost-effective care (SBP3) | 7.07 ± 0.91 | 7.39 ± 0.59 | 0.06 | 0.35 |
| Systems-Based Practice: Transitions patients effectively within & across health delivery systems (SBP4) | 7.56 ± 0.71 | 7.71 ± 0.72 | 0.36 | 0.21 |
| Practice-Based Learning: Monitors practice with a goal for improvement (PBLI1) | 7.15 ± 1.04 | 7.68 ± 0.72 | 0.01 | 0.51 |
| Practice-Based Learning: Learns & improves via performance audit (PBLI2) | 7.05 ± 0.77 | 7.41 ± 0.63 | 0.02 | 0.47 |
| Practice-Based Learning: Learns & improves via feedback (PBLI3) | 7.24 ± 0.83 | 7.66 ± 0.69 | 0.02 | 0.51 |
| Practice-Based Learning: Learns & improves at the point of care (PBLI4) | 7.12 ± 0.93 | 7.56 ± 0.78 | 0.02 | 0.47 |
| Professionalism: Has professional & respectful interactions with patients, caregivers & members of the interprofessional team (PROF1) | 7.41 ± 0.95 | 8.00 ± 0.87 | 0.01 | 0.62 |
| Professionalism: Accepts responsibility & follow through on tasks (PROF2) | 7.24 ± 0.99 | 7.93 ± 0.98 | 0.002* | 0.70 |
| Professionalism: Responds to each patient's unique characteristics & needs (PROF3) | 7.27 ± 0.81 | 7.88 ± 0.87 | 0.002* | 0.75 |
| Professionalism: Exhibits integrity & ethical behavior in professional conduct (PROF4) | 7.44 ± 0.90 | 7.95 ± 0.89 | 0.01 | 0.57 |
| Interpersonal Communication: Communicates effectively with patients & caregivers (ICS1) | 7.29 ± 0.84 | 7.85 ± 0.82 | 0.003 | 0.67 |
| Interpersonal Communication: Communicates effectively with interprofessional teams (ICS2) | 7.22 ± 1.04 | 7.90 ± 0.77 | 0.001* | 0.65 |
| Interpersonal Communication: Appropriate Utilization & completion of health records (ICS3) | 7.46 ± 0.67 | 7.71 ± 0.68 | 0.11 | 0.37 |

*Bonferroni corrected statistical significance level p = 0.002
**Cohen’s d < 0.1 (trivial effect size); Cohen’s d between 0.1-0.3 (small effect size); Cohen’s d between 0.3-0.5 (moderate effect size) Cohen’s d > 0.5 (large effect size)
